# Molecular signaling and targets from itch: lessons for cough

**DOI:** 10.1186/1745-9974-9-8

**Published:** 2013-03-06

**Authors:** Pamela Colleen LaVinka, Xingzhong Dong

**Affiliations:** 1 The Solomon H. Snyder Department of Neuroscience, Center for Sensory Biology, Johns Hopkins University School of Medicine, 725 N Wolfe Street, Baltimore, MD, 21205, USA; 2 Howard Hughes Medical Institute, Johns Hopkins University School of Medicine, Baltimore, MD, 21205, USA

**Keywords:** Itch, Cough, Skin, Airway, C-fibers, A-fibers, Mast cells, TRPs, GPCRs, Inflammation

## Abstract

Itch is described as an unpleasant sensation that elicits the desire to scratch, which results in the removal of the irritant from the skin. The cough reflex also results from irritation, with the purpose of removing said irritant from the airway. Could cough then be similar to itch? Anatomically, both pathways are mediated by small-diameter sensory fibers. These cough and itch sensory fibers release neuropeptides upon activation, which leads to inflammation of the nerves. Both cough and itch also involve mast cells and their mediators, which are released upon degranulation. This common inflammation and interaction with mast cells are involved in the development of chronic conditions of itch and cough. In this review, we examine the anatomy and molecular mechanisms of itch and compare them to known mechanisms for cough. Highlighting the common aspects of itch and cough could lead to new thoughts and perspectives in both fields.

## Introduction

Over 350 years ago, the German physician Samuel Haffenreffer defined itch, or pruritus, as an “unpleasant sensation that elicits the desire or reflex to scratch.” Why is such an unpleasant sensation needed? Itch causes the protective mechanism of scratching. The physical act of scratching dislodges the irritant, such as an insect or poisonous plant, from the skin. Therefore itch results in the removal of a harmful stimulus. Scratching may not just be protective though. The actual act of scratching can result in mechanical pain, which helps to suppress the unpleasant itchy sensation [1,2]. Therefore scratching can both help remove an irritant and try to suppress the itch resulting from it.

Although the skin is the primary site for the generation of itch, itchiness can also be felt in mucosal surfaces. So perhaps itch is felt in the airway and since we cannot scratch our airway, it is reasonable to think that the main response to airway itch would be to cough. Coughing helps to clear irritants and evoke itch-suppressing mechanical pain in the airway, similar to scratching itchy skin. Coughing is most easily evoked from stimulation of the larynx, trachea, and larger bronchi and many types of stimuli can result in cough, not just itch [3]. The cough reflex results in the removal of foreign material from the large bronchi with successive coughs forcing the foreign material or secretion through the smaller bronchi towards the trachea so the irritants can be expelled [4,5].

In this review, we will talk about the mechanisms of itch and cough and the similarities found between the pathways. The parallels between itch and cough can perhaps lead to new perspectives and ideas in ways to test the respective pathways and how they may possibly work.

## Review

### Primary sensory fibers of itch and cough pathways

**Figure 1:**
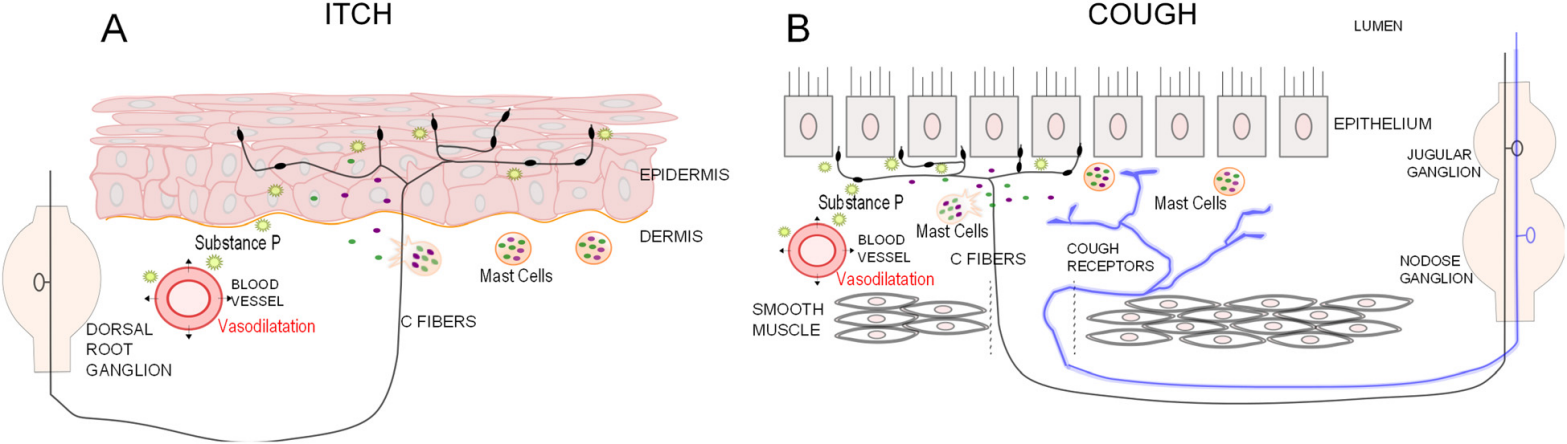
**Primary sensory neurons of itch and cough. A**. Itch is primarily sensed through the epithelium by unmyelinated C-fibers, whose cell bodies are in the dorsal root ganglion. Degranulation of mast cells release inflammatory mediators that can activate C-fibers, which secrete neuropeptides, such as Substance P. Secretion of Substance P results in inflammation and vasodilatation, which sensitizes nerves. **B**. Cough results from activation of myelinated cough receptors and unmyelinated C-fibers, whose cell bodies are in the jugular and nodose ganglia. Extensive C-fiber endings are found under the airway epithelium while cough receptors endings terminate in the mucosa between the epithelium and smooth muscle. Degranulated mast cells release mediators that activate C-fibers, causing release of Substance P. Substance P release results in inflammation, vasodilatation and sensitization of nerves.

Before looking at the specifics of a sensory system, we should first look at the basic anatomy that transmits the sensory information. Figure 1 illustrates sensory fibers that are primarily responsible for itch and cough. Primary sensory neurons in dorsal root ganglia (DRG) play an essential role in generating itch by detecting itch stimuli through their peripheral axons in the skin and sending signals to the spinal cord via their central axons [6]. In the cough pathway, the cough sensory afferent fibers terminate in or under the airway epithelium with their cell bodies located in the vagal nodose or jugular ganglia [4]. The nodose ganglia are embryologically derived from the epibranchial placodes while the jugular ganglia originate from the neural crest [7]. Interestingly, the DRG sensory neurons also originate from the neural crest [8]. Sensory neurons that play a role in itch or cough can be classified into two distinct fibers, the thinly myelinated Aδ-fiber and the unmyelinated C-fiber.

#### Myelinated Aδ-fibers

##### Itch

In early investigations into itch, it was suggested that there was two subjective components to itch. First a ‘pricking’ itch was felt, followed by a ‘burning’ itch. The ‘pricking’ itch was sensed via the myelinated sensory fibers and abolished when these myelinated fibers were blocked [9]. It has been shown in studies involving the known pruritogen cowhage (Mucuna pruriens), that mechanosensitive Aδ-fibers play a role in itch. Cowhage causes intense itching when injected into the skin [10]. Psychophysical human studies show decreased itch from cowhage upon blocking of myelinated A fibers. In monkeys, cowhage activates mechanosensitive A fibers while some mechanoinsensitive A fibers are activated by another pruritogen, histamine [11].

##### Cough

Myelinated Aδ-fibers play a role in the generation of cough. The Aδ-fibers are responsible for the violent, sudden cough that occurs upon aspiration [12]. These myelinated vagal afferent cough fibers are sensitive to acid and mechanical stimulation but are relatively insensitive to capsaicin and the effects of anesthesia [13]. In dogs, when myelinated nerve conduction is blocked by vagal cooling, mechanically and chemically stimulated cough is inhibited [14]. Single fiber vagal recordings of afferent Aδ-fibers in the guinea pig trachea activate upon exposure to known cough-evoking hypotonic, hypertonic, and low-chloride stimuli [15]. Vagal Aδ-fibers also show sensitivity to very low levels of touch stimulus, which can trigger cough [16].

Different types of Aδ-fibers are involved in the regulation of breathing and cough. These different myelinated vagal sensory fibers include rapidly adapting receptors (RARs), slowly adapting receptors (SARs) and the cough receptors. The RARs and SARs are differentiated on their ability to adapt to lung inflation, either rapidly or slowly [17-19]. RARs and SARs are thought to play a modulatory, synergistic role in the cough reflex but are not responsible for initiating cough [13,20]. The myelinated vagal fiber thought to be responsible for initiating cough is the touch sensitive Aδ-fiber or cough receptor. The cough receptor cell bodies are mostly located in the nodose ganglia with axons terminating in a circumferential pattern between the epithelium and smooth muscle in the mucosa of the guinea pig trachea [21]. The cough receptors are very sensitive to punctate mechanical stimuli and changes in pH in conscious and anaesthetized animals [22]. Up till recently, the cough receptor was included in the RAR fibers, until Canning et al. distinguished this subset in their 2004 paper. Cough receptors have several unique qualities that discriminate them from being classified as RARs or SARs. Cough receptors do not show a response to changes in lung volume like RARs and SARs and are primarily located in the extrapulmonary airways, rather than the intrapulmonary airways [22]. These afferent myelinated fibers project to the trachea via the recurrent laryngeal nerves and when these nerves are cut, coughing is abolished [22]. The nodose vagal afferent fibers conduct action potentials at 3–5 m/s and lack TRPV1 receptors, which is why they are relatively insensitive to capsaicin [22,23].

The role of myelinated fibers in cough is much more defined and explored when compared to myelinated fibers’ role in itch. What is interesting in both is that these myelinated fibers are not solely responsible for the genesis of itch or cough. The myelinated A-fibers interact with unmyelinated C-fibers to respond to irritating stimuli, resulting in itch or cough. In the skin, cowhage causes itch through activation of both myelinated and unmyelinated fibers [11,24]. In the airway, activation of C-fibers results in many of the same reflexes (cough, bronchoconstriction) as activation of myelinated fibers, so the reflexes are likely a result of both types of sensory fibers [25]. In both itch and cough, C-fibers play a role in setting the threshold, controlling the sensitivity of the system.

#### C fibers

##### Itch

Itch is primarily mediated by slower conducting C-fibers innervating the dorsal horn of the spinal cord. Early itch studies used spicules of cowhage to show that the strongest itch is felt in the dermo-epidermal juncture area, the area where unmyelinated sensory fibers innervate [10,26]. Five percent of the C-fibers innervating the skin react to puritogenic application, with distinct populations of C-fibers corresponding to itch in individual nerve C-fiber recordings in humans [24,27]. Itch sensing C-fibers slowly conduct action potentials (0.5 m/s) and innervate large territories of skin [27]. When C-fibers are desensitized with pretreatment of capsaicin, itch is greatly reduced or blocked [28].

However, not all C fibers respond equally to all pruritogens. C-fibers can be divided into subcategories according to their response to various stimuli and these subcategories respond differently to different pruritic stimuli. C-fibers classified as mechanical and heat responsive (CMH) have been shown to respond to cowhage and histamine [29]. Mechanically-insensitive C-fibers that respond to histamine (CMiHis+) have a preferential, not exclusive, activation to pruritic substances such as histamine and prostaglandin E(2) (PGE2) and substances that activate more of these specific CMiHis+ fibers are considered more potent pruritic agents [19]. Microneurography recordings of C-fibers in human volunteers show that cowhage activates mechanosensitive C-fibers while histamine activates mechano-insensitive units. Capsaicin activates fibers in both classes [24]. So cowhage induces itch through a different set of C-fibers than histaminergic itch. The activation of two different subsets of C-fibers shows that there are multiple pathways for pruritus, changing according to the stimulus.

##### Cough

C-fibers are also involved in the generation of cough. It is thought that C-fibers are important to a second type of cough, which is used to get rid of an itchy feeling in one’s throat. This feeling is more typical of chronic cough [12]. Cough is generated when vagal C-fibers and myelinated cough receptors are activated [20]. The C-fibers form a loose network in the airway mucosa with branches found in deeper lung structures [7]. The majority of C-fibers innervating the airway arise from the vagus nerve, however there is also some innervation by spinal afferent C-fibers whose cell bodies are in the DRG [30]. There are two sets of vagal C-fibers, the bronchial C-fibers which innervate the large airways and the pulmonary C-fibers which innervate the smaller, peripheral lung tissue [30]. Collectively these C-fibers are the bronchopulmonary C-fibers and can be activated using a variety of chemical stimuli, including capsaicin, bradykinin and protons. Inhalation of these chemicals such as capsaicin in humans causes an itchy feeling in the airway and evokes cough [12,20,31,32]. In single fiber recordings, all vagal C-fibers tested respond to capsaicin and capsaicin-sensitive C-fibers are found in both the nodose and jugular ganglion [7,16]. However, in dogs and rats, rapid shallow breathing is caused by C-fiber activation, but not cough, showing that there can be species differences [3,14,33]. In fact, C-fiber activation can inhibit mechanically induced cough [14]. These inhibitory C-fibers are thought to be the C-fibers with cell bodies in the nodose ganglion. Indeed, activation of nodose C-fibers with adenosine reduce citric acid evoked cough [20]. This implies that the jugular ganglia C-fibers are the ones playing an excitatory role in cough. The differences seen in C-fibers originating from the nodose ganglia versus C-fibers originating from the jugular ganglia are evidence of a multi-faceted cough reflex with many ways to fine-tune a response. However, the fact that many C-fiber activators cause cough in awake guinea pigs and humans, speaks to their role in generating cough.

Anatomically, the chemosensitive C-fibers extend numerous terminals superficially into the airway epithelium, placing them in an ideal position to react to inhaled chemical irritants [34]. Labeling studies in guinea pigs show C-fibers terminating underneath the airway epithelium, with swelling suggestive of the presence of synaptic vesicles filled with Substance P (SP), Calcitonin gene related peptide (CGRP) and Neurokinin A [21]. When the fibers innervating the tracheal epithelium are analyzed, nearly all C-fibers have cell bodies in the jugular ganglion and 60% are containing fibers [32,35]. These jugular C-fibers promote coughing [20]. No peptide positive fibers are found in the nodose ganglion [32,36]. So within the C-fibers there are two subsets, peptidergic (in jugular ganglion) and non-peptidergic (in nodose ganglion) [37]. However, the C-fibers that innervate the lungs (below the trachea) originate from both ganglia, with over 60% of C-fibers in the lungs found in the nodose ganglion [7].

Many argue that direct cough is caused by the touch sensitive Aδ-fibers and that C-fibers cause cough by indirect mechanisms. This is shown by C-fiber evoked cough’s sensitivity to anesthesia. Anaesthetized animals often do not cough upon application of C-fiber stimulants but cough when awake. Stimulation of bronchopulmonary C-fibers with chemical stimuli can result in bronchoconstriction and mucous production, both of which can cause cough [38]. Stimulated C-fibers release which mediates nitric oxide and results in an increase in fluid in airways, activating RARs and causing cough [39]. It was found that pretreatment with a C-fiber stimulant, while not evoking cough alone, decreased the cough threshold for RAR and cough receptor stimulation. Conversely, desensitizing C-fibers with capsaicin application, led to a decrease in coughing upon myelinated cough fiber activation [40]. These findings suggest a synergistic central interaction between C-fibers and myelinated RARs and cough receptors.

With the knowledge that C-fibers are involved in both itch and cough, it is likely that there are similarities to be found in activation of these sensory neurons. Large territories of innervation by C-fibers are seen in both the skin and lung. The jugular ganglion is derived from the neural crest, just like the dorsal root ganglion. With similar embryonic starts, these fibers may share many characteristics. Neural crest derived vagal nerves are seen innervating the large extrapulmonary airways while placodal nerves are seen innervating deeper lung tissue leading to speculation that the more superficial neural crest derived vagal nerves are responsible for reacting to external environmental stimuli [7,37]. This parallels the more superficial termination of C-fibers in the skin that are thought to be responsible for itch [41].

Knowing that itch and cough are mediated by similar sensory neurons, the specifics of activating these fibers can be examined and compared. Two types of receptors are activated on sensory fibers, ionotropic and metabotropic. In both of these categories, itch and cough work through the same receptors in multiple instances. Table 1 lists some of the known pruritic and tussive agents and modulators as well as their pathways.

**Table 1:** Activators and modulators of itch and cough pathways

**Agent**	**Pathway**	**Evoke Itch**	**Evoke Cough**
Bradykinin	B1, B2	Weakly, modulator	Yes, modulator
Capsaicin	TRPV1	superficial application, Yes, modulator	Yes
Oxidative	TRPA1	Yes	Yes, modulator
Cowhage	PAR2, PAR4	Yes	Yes
Histamine	H1, H4	Yes, modulator	Modulator
Proteases	PAR1, PAR2, PAR4	Yes	Modulator
Serotonin	5HT2, 5HT3	Yes	Modulator
Substance P	NK1, NK2	Yes	Modulator

### Ionotropic receptors of itch and cough

#### TRPV1

##### Itch

The transient receptor potential, vanilloid 1 (TRPV1) receptor is a membrane bound, ligand gated channel. It is a six transmembrane spanning protein that undergoes a conformational change upon binding of a ligand, allowing cations into the nerve and resulting in activation of primary sensory neurons [42]. The role of TRPV1 has often been explored using one of its most famous ligands, capsaicin. If capsaicin is applied in a punctuate manner to the epidermis, it causes itch [43]. TRPV1 are expressed on a subset of C-fibers and repeated application of capsaicin will desensitize these C-fibers. Desensitizing C-fibers help reduce the itch induced by histamine [28]. When TRPV1 receptors were blocked by capsazepine, histamine evoked currents were reduced in sensory neurons. Similarly, histamine failed to activate TRPV1^−/−^ neurons [44]. Therefore, TRPV1 plays an important role in histamine-dependent itch. Mice deficient in Pirt, a TRPV1 modulator, have decreased scratching in response to histamine, chloroquine, and ET-1, implicating TRPV1 as an important component in multiple itch pathways [45].

While TRPV1 can be directly activated by capsaicin, its main role is functioning downstream of many pruritogens. Pruritogens activate G protein coupled receptors (GPCRs) that result in intracellular cascades that can activate TRPV1 [44,46]. GPCR activation results in production of phospholipase C (PLC) beta3, an intracellular mediator that activates TRPV1 upon histamine application [44]. PLCbeta3 also mediates serotonin-evoked itch [46]. TRPV1 can also be activated by diacylglycerol (DAG) which is produced when PIP2 is hydrolyzed by PLC [46,47]. Expression of TRPV1 and phosphorylated TRPV1 receptors increase in atopic dermatitis (AD) mouse models lesions [48]. TRPV1 contributes to skin inflammation by causing release which leads to upregulation of SP’s receptor, neurokinin 1 (NK1). Increased levels of NK1 expression is seen in lesions from AD models [48,49]. Blocking TRPV1 stops the upregulation of NK1 receptors and decreases bouts of scratching [48].

##### Cough

TRPV1 is also thought to be a strong effector of the cough reflex in response to many different stimuli [50]. TRPV1 is found in both vagal ganglia as well as throughout the airway [51,52]. Airway mucosal biopsies from patients suffering from chronic cough showed a fivefold increase in TRPV1 expression [53].

Capsaicin is a commonly used tussive agent and resiniferatoxin, a strong TRPV1 agonist, causes cough by direct activation of TRPV1 [54]. PGE2 and bradykinin, which are known to cause cough, depolarize vagal sensory neurons through activation of TRPV1 [50]. Citric acid evoked cough works through activation of TRPV1 and antagonizing the receptor with capsazepine and ruthenium red reduces citric acid cough [55,56]. Anandamide has been shown to activate nodose ganglion cells and induce cough in guinea pigs through a TRPV1-dependent mechanism [57].

#### TRPA1

##### Itch

TRPA1 is a strong noxious sensor due to reactive cysteines that can form covalent bonds with multiple chemical compounds [58,59]. TRPA1 has been shown to be important to histamine-independent itch. The GPCRs MrgprA3 and MrgprC11 are activated by chloroquine and BAM8-22 respectively, both of which cause scratching [60]. When TRPA1 is blocked or deleted, itch caused by BAM and chloroquine is drastically reduced [61]. This indicates TRPA1 is activated downstream by MrgprA3 and MrgprC11. Whereas TRPV1 is activated by G-alpha activating the PLC pathway, TRPA1 is shown to interact with the G-Beta-Gamma subunit directly [61]. It has been recently shown that oxidative challenges (H_2_O_2_ injections) can cause scratching, seemingly as a result of itch. The mechanism was shown to be dependent on TRPA1, not TRPV1 [62]. This is important as oxidative stress is involved in diseases causing chronic itch [63].

##### Cough

TRPA1 is found in TRPV1^+^ vagal sensory neurons innervating the airway and accordingly, cinnamaldehyde (TRPA1 agonist) stimulates capsaicin (TRVP1 agonist) sensitive neurons [64]. Since TRPA1 is found in the airway and reacts to many chemicals, it is a good candidate for an environmental sensor that can activate cough. TRPA1 has been shown to be an oxidant sensor in murine airway neurons [65,66]. Indeed, multiple TRPA1 ligands are found to evoke cough in guinea pigs and humans [67]. TRPA1 agonists stimulate jugular C-fibers innervating the trachea and TRPA1 mediates irritation induced by chemicals found in cigarette smoke and air pollution (eg acrolein and crotonaldehyde) [68,69]. Interestingly, TRPA1 has been shown to evoke cough independent of TRPV1 as TRPV1 antagonists could not prevent acrolein evoked cough. However, desensitization of the C-fibers that contain both TRPA1 and TRPV1 did abolish TRPA1 cough [70].

### Inflammatory mediators

Activation of TRP channels leads to release of inflammatory neuropeptides from C-fibers. These neuropeptides include the tachykinins (Substance P, neurokinin A, neurokinin B) and calcitonin gene related peptide (CGRP) [71]. Other inflammatory chemicals, such as bradykinin, may also be released. Neurogenic inflammation has been shown to play roles in both chronic itch and chronic cough.

#### Substance P

##### Itch

Application of SP to the skin in humans causes itch which is prevented by antihistamines and so is thought to be histamine dependent [72]. This collaborates with the fact that depleting mast cells, a primary source of histamine, with compound 48/80 decreased the itching, wheal and flare response from SP intradermal injections in human volunteers [73]. Substance P is thought to mediate itch through activation of the neurokinin (NK) receptors. After skin-scratching stimulation, NK1 is upregulated in the epidermal keratinocytes and Substance P is depleted from sensory fibers, indicating secretion of SP into the surrounding cells [74]. It was found that NK1 antagonists inhibit scratching in a dermatitis model [75]. Clinically, patients with chronic pruritus have reduced itching upon using a NK1 antagonist [76].

##### Cough

Substance P results in plasma extravasation in the airways, which can activate RARs [77]. In vivo, Substance P only causes weak and variable cough in guinea pigs [78]. Furthermore, nodose neurons from healthy guinea pigs show no reaction to Substance P application. However, once inflammation is induced by antigen inhalation, 80% of nodose neurons respond to Substance P. Airway inflammation unmasks the Substance P response that is shown to be mediated by NK2 receptors [79]. Increased levels of Substance P are found in the nasal epithelial cells of patients with cough hypersensitivity and chronic cough. This correlates with increased levels of nitrosative stress, which enhances Substance P secretion [80]. Increased Substance P is also found in the plasma of chronic cough sufferers [81].

#### Bradykinin

##### Itch

Application of bradykinin to the skin evokes feelings of weak itch and pain in healthy skin. However, pruritic inflammation can change the effect of bradykinin. If bradykinin is applied to itchy inflamed skin, like lesions in AD patients, much more intense itch is felt. Itch evoked by bradykinin is histamine-independent [72]. When inflammation is induced by complete freunds adjuvant (CFA), subsequent application of bradykinin causes robust scratching. This behavior is mediated by the kinin B1 receptor [82]. Both kinin receptors, B1 and B2, are shown to contribute to itch. This is proven by the reduced scratching of B1 and B2 knockout mice to different pruritic stimuli. B1 and B2 antagonists have similar effects [83]. Data suggests that pruritic responses are mediated mostly by B2 receptors. Antagonists of kinin (B1 and B2) receptors help reduce itch from PAR2 agonists, implying that kinin receptors are activated downstream from PAR2 and can mediate protease evoked itch also [83]. It has been shown that patients taking angiotensin converting enzyme (ACE) inhibitors for hypertension, occasionally suffer from pruritus as a side effect [84]. ACE inhibitors lead to increased levels of bradykinin, as ACE degrades bradykinin usually. The increased levels of bradykinin are thought to cause the pruritus, which is usually a precursor to a much more dangerous side effect, angioedema [84,85].

##### Cough

Bradykinin is endogenously produced, with airway inflammation causing an increase in levels [86]. Bradykinin can depolarize human, guinea pig and mouse vagal afferents, activating C- and Aβ-fibers in the jugular ganglion as well as nodose C-fibers [50,87]. In humans, bradykinin causes cough and bronchoconstriction [88,89]. Bradykinin also evokes cough in guinea pigs [16]. Activation is thought to occur through the B2 receptors in the guinea pigs and humans but the B1 receptor is also involved in mice, showing species differences [16,50,87]. B2 receptors are found in the vagal nodose ganglion of rats and humans [90]. Bradykinin is also implicated in sensitizing the cough reflex, enhancing cough response to citric acid [78]. Patients taking ACE inhibitors (which normally degrades bradykinin) often develop chronic cough [91]. In fact, patients often stop taking the ACE inhibitor due to the chronic cough that develops. Bradykinin’s tussive effects are tied to activation of TRPV1 and TRPA1. Bradykinin’s activation of B2 receptors works through the Gq/11 transducer, which results in PLC and possible stimulation of TRPV1 [92,93]. Antagonists of either channel alone partially inhibited in vivo and in vitro responses of vagal neurons to bradykinin. Applying a TRPV1 antagonist along with a TRPA1 antagonist completely prevented the effects of bradykinin [50].

### Mast cell mediators

Mast cells are found in close contact to nerves, allowing them to easily affect sensory perception [94]. Mast cells play important roles in both itch and cough, especially in chronic conditions. Scratching the skin of mice causes significant mast cell degranulation within minutes [74]. In atopic dermatitis mouse models, increased levels of degranulated mast cells are found [48]. In patients diagnosed with chronic nonproductive cough (CNPC), bronchoalveolar lavage (BAL) showed increased numbers of inflammatory cells and airway inflammation when compared to controls [95]. Elevated levels of mast cells were found in BAL samples in patients with chronic cough [96]. Upon activation, mast cells degranulate and release biologically active mediators. These mediators include histamine, serotonin, and proteases [97]. We will discuss the roles of these mast cell mediators in itch and cough below.

#### Histamine

##### Itch

It is well established that introduction of histamine to human skin causes itch [98,99]. Histamine also evokes scratching in a dose-related manner in mice [100]. Spinothalamic neurons that respond to itch-evoking histamine but not pain-evoking mustard oil were found in the cat, implicating an itch specific pathway [101]. The similarities and differences between itch and pain have been discussed in other reviews and will not be covered here [41].

Histamine is naturally produced by the body from the amino acid histadine, with mast cells being one of the main sources of histamine in the body [102,103]. There are four subtypes of histamine receptors, H1-H4 [104]. The H1 receptor plays a starring role in evocation of itch via histamine [105]. Antihistamines that block the H1 receptor have proven helpful in the treatment of uticaria (hives), decreasing itching and reducing size of wheals and flares [102]. Histamine receptors are GPCRs, activating an intracellular signaling network that results in activation of ionotropic receptors, producing an action potential. Therefore, histamine evokes currents only when the H1 receptor is coexpressed with the ionotropic TRPV1 receptor [44]. The H1 receptor is coupled with G_q_ proteins and activates PLC [106]. Specifically H1 receptors are mediated through PLCβ3, which activates TRPV1 [44,107]. Histamine is also shown to activate TRPV1 via PLA2 [44]. Inhibiting PLA2 stops histamine-induced Ca^2+^ influx in sensory neurons [108]. H4 receptors are also implicated in itch. Histamine causes itching in atopic dermatitis through activation of the H4 receptor [102]. Patients with AD are also more sensitive to histamine, showing increased responses in their skin lesions [109]. H4 agonists cause scratching upon injection and H4 antagonists decrease itching and inflammatory and pruritic symptoms in AD mouse models [110-112].

##### Cough

Histamine also plays an important role in modulating cough sensitivity. Histamine does not directly cause cough as its application does not result in action potentials in guinea pigs’ isolated vagal preps [16,32]. In fact, application of histamine to vagal nodose neurons only results in a small membrane depolarization [113]. However, histamine does sensitize vagal bronchopulmonary C-fibers’ response to capsaicin and mechanical stimulation [114]. Antagonizing histamine via the H1 receptor significantly attenuated citric acid evoked cough [115]. Causing increased cough sensitivity can lead to chronic cough and chronic cough sufferers do have elevated levels of histamine in their sputum and lungs [96,116]. Eosinophilic bronchitis, a disease often causing chronic cough, increases levels of histamine in patients’ sputum [117]. Human studies using ultrasonically nebulised distilled water (UNDW) to evoke cough show reduced UNDW responses when loratadine, an H1 antagonist, is given to patients suffering from chronic dry cough. This reduction implicates a role for histamine in chronic cough conditions [118].

#### Serotonin

In rodents, a main source of serotonin is mast cells [119]. While human mast cells can synthesize and secrete serotonin and may do so in inflammatory conditions, this is not thought to be the primary source of serotonin [120]. Serotonin can be found in platelets and neuroepithelial bodies, which are innervated by vagal sensory neurons, in the airway and thus still play a role in cough evocation [121,122].

##### Itch

Serotonin (5-HT) causes scratching when injected into the faces of rats [123]. Topically applying serotonin to the back of rats also results in scratching and activates DRG neurons [124]. Mice also scratch in a dose-related manner to 5HT [100]. In a dry skin chronic itch model, a 5HT antagonist (ketanserin) significantly decreases bouts of scratching in mice [125]. This implies a role for 5HT in chronic itch and patients suffering from eczema and psoriasis do show increased expression of 5HT [126,127]. However, in human studies, injection of 5HT causes mixed feelings of itch and pain. The mixed sensations are felt through activation of a subset of cutaneous C fibers [128]. Itching from intradermal 5HT injections is thought to be partially mediated by the metabotropic 5-HT2 receptor as agonists of this receptor induce scratching and antagonists reduce scratching [129]. The ionotropic 5-HT3 receptor may also play a role in itch as antagonists of the 5-HT3 receptor may be effective in treatment of opioid-induced pruritus [130].

##### Cough

Serotonin stimulates respiratory reflexes [131]. In dogs, phenylbiguanide, a 5HT receptor agonist, activates bronchial C-fibers [132]. Nodose ganglia C-fibers respond to serotonin [93]. Specifically, serotonin activates the ionotropic serotonin receptor 5HT3 in rabbit nodose ganglion neurons [133,134]. Triggering 5HT3 receptors leads to membrane depolarization of most small diameter neurons in the vagal ganglia [135]. The guinea pig also shows activation of the 5HT3 receptor on intrapulmonary nodose C-fibers [93]. Interestingly, the jugular ganglion C fibers in guinea pigs do not respond to 5HT [136]. However, 5HT does stimulate jugular ganglion C fibers in mice, possibly through a metabotropic 5HT receptor [137]. It is possible the metabotropic 5HT2A receptor might be involved, because in mouse tracheal preps, serotonin causes tracheal muscle contraction via the 5HT2A receptor [119]. This contrasts with activation of the nodose C fibers of mice, which is mediated by the ionotropic 5HT3 receptor [137].

#### Proteases

##### Itch

Endogenous serine proteases, including tryptase and trypsin, cause itch by activation of the Protease activated receptor (PAR) family. These GPCRs are activated when cleavage of the NH2 terminus of the PAR receptor results in a tethered self-activating ligand [138]. There are four members of the PAR family, PAR1-4 [139]. PAR2 is involved in itch. PAR2 is a seven-transmembrane GPCR that is proteolytically activated by trypsin and the agonist SLIGRL [140]. When SLIGRL is injected into the skin of mice, robust scratching is shown [141,142]. SLIGRL activation of PAR2 releases PGE2 from keratinocytes, whose secretion enhances scratching behavior [143]. DRG neurons show PAR2 cells that coexpress with Substance P and CGRP. When mast cells release tryptase, it activates PAR2. PAR2 activation results in release of the coexpressed neuropeptides, causing inflammation [144]. This activation cascade is thought to play a role in trypsin induced scratching as well. Trypsin activates PAR2 on mast cells, which results in release of SP and CGRP and scratching. Data supports this by showing depletion of mast cells prevented trypsin induced scratching [145]. In chronic itch conditions such as atopic dermatitis, increased expression of PAR2 was found on primary afferent sensory nerves as well as increased levels of tryptase [146]. Dry skin mouse models exhibited sensitization to PAR2 agonists and a PAR2 antibody helped reduce scratching [125]. PAR2 itch and neuron sensitization is thought to occur through interaction with TRP channels, like TRPV1. Deletion of TRPV1 or introduction of a TRPV1 antagonist prevents scratching from trypsin injection [145]. PAR2 has also been shown to coexpress with TRPV4 and TRPA1 [147,148]. PAR2 pathways are histamine independent because antihistamines did not help reduce itch in atopic dermatitis or mice injected with SLIGRL [141,146]. PAR4 has also been shown to elicit scratching in mice upon activation [100]. PAR2 and PAR4 work through the G_q_ protein and Ca^2+^ signaling [27]. The active component in cowhage, mucunain, has been shown to be a ligand for PAR4 in addition to PAR2 [149].

##### Cough

Proteases and their receptors also play a role in cough and airway inflammation. In vagally innervated lung preparations, trypsin-like protease thrombin has been shown to activate bronchopulmonary C-fibers by activating PAR1 [30]. PAR2 has been implicated in airway inflammation also. PAR2 can be found in the airway epithelial cells and smooth muscle of guinea pigs and PAR2 agonists can cause bronchoconstriction [150]. Airway hyperactivity to inhaled stimulants was decreased in PAR2 knockout mice and increased in mice overexpressing PAR2 when compared to wildtype controls [151]. Patients suffering from bronchitis were found to have increased expression of PAR2 [152]. This finding is supported by the knowledge that mucosal inflammation, a symptom of bronchitis, causes an upregulation of PAR2 in the airway epithelium; as was found in the skin in reference to itch, activation of PAR2 by mast cell tryptase causes release of PGE2 in the airway [153]. Inhalation of PGE2 has been shown to cause cough in vivo and to sensitize the pulmonary C fiber cough reflex [89,154].

Activation of PAR2 does not evoke cough directly as data shows PAR2 agonist trypsin does not cause action potentials in C-fibers innervating the trachea or bronchi [155]. Activating PAR2 leads to PGE2 release though, which has been shown to cause cough in vivo. Also PAR2 plays a role in potentiating cough by sensitizing cough evoked by TRPV1 stimulation [156]. PAR2 was shown to coexpress with TRPV1 and sensitize the receptor through phosphorylation by the protein kinase C pathway [156,157].

Doubt on PAR2’s role in itch was recently shown in a 2011 paper by Liu et al. It was shown that the PAR2 agonist SLIGRL activates a member of the Mrgpr family, MrgprC11 and it is through the activation of this receptor, that SLIGRL evokes itch [158]. This study also suggested that trypsin induced itch did not act through PAR2 or MrgprC11. Rather trypsin could act through another PAR or an unknown subset of fibers. This broadening of our knowledge of itch reminds us that cough could be very similar. While PAR2 itself does not seem to activate C fibers and induce cough, an unknown subset of C fibers could be activated by the PAR2 agonists, similar to the activation of MrgprC11 by SLIGRL.

#### Gastrin-releasing peptide receptor

Recently, evidence for itch specific neurons called gastrin-releasing peptide receptor (GRPR) has been published. The discovery of the pruritic role of these neurons has opened new paths of discovery. With the idea of itch and cough being similar in many respects, it would be remiss of us not to compare the role of GRPR in both conditions.

##### Itch

Gastrin-releasing peptide (GRP) is the mammalian homologue of the amphibian neuropeptide bombesin. GRP was found on peptidergic unmyelinated small to medium sized DRG neurons with 80% of GRP^+^ neurons also expressing TRVP1 receptors [142]. In GRPR knockout mice, scratching from histamine, SLIGRL and chloroquine induced itch was reduced [142]. GRPRs are found in lamina I of the dorsal horn and when an GRPR agonist was injected intrathecally, bypassing peripheral activation of the skin, scratching was exhibited, supporting the idea of GRPR cells being itch specific [142]. When GRPR spinothalamic cells were ablated using bombesin-saporin, reduction of scratching from pruritogenic (both histamine-dependent and histamine-independent) stimulation was practically extinguished [159]. This included scratching induced by 5HT and compound 48/80, a mast cell degranulator.

##### Cough

Bombesin induces bronchoconstriction in guinea pigs airways, an effect that is absent if the trachea or bronchi was stripped of its epithelium [66]. In rats, GRP and bombesin exposure cause an increase in rapid, shallow breathing characteristic of activation of C-fibers. In fact, GRP and bombesin increase the pulmonary chemoreflex response to capsaicin. This potentiation is blocked when pulmonary C-fiber conduction is not allowed [160]. All three types of bombesin-like peptide receptors are found in human lung tissue, specifically in human bronchial epithelial (HBE) cells [161]. Having the receptors located in the airway epithelium, close to where C-fibers terminate, implies that GRP could play a regulatory role on the C-fibers involvement in the cough reflex.

### Conclusions and future directions

The field of itch research has only started to be explored, with much expansion seen over the last couple of years. There are still many challenges that need to be overcome in the itch field. Recently, many novel itch receptors and pruritogens have been identified, however, our knowledge on how they function or mis-function during chronic itch conditions is still limited. Is there a common downstream factor which is required for most, if not all, itch signaling pathways? If such a factor is discovered, it will most likely be found using animal studies. Therefore as our knowledge of itch expands, these findings should also be validated in human studies. This will require extensive collaboration between basic researchers and clinicians, eg dermatologists, which can be logistically difficult. Another challenge is that while most major pharmaceutical companies are developing anti-pain drugs, many companies do not realize that chronic itch is a major clinical problem. Without industrial collaboration, it is difficult for basic research laboratories to conduct large scale screens to identify itch blockers. Pharmaceutical companies need to be convinced that development of anti-chronic itch drugs will have large market values.

As our anatomical knowledge of itch grows, it is obvious that the sensory Aδ- and, more importantly, C-fibers play a pivotal role in itch perception. Tied closely to activation of these sensory fibers is neurogenic inflammation, which involves release of inflammatory agents like SP and bradykinin as well as products of mast cells, all which result in itch, flares, wheals, and can easily become chronic conditions. All of these individual factors also play roles in cough and the similarities between itch and cough in sensing irritants from environment can be seen. Cough has an additional factor to incorporate though, movement. While the end result of itch is scratching, the muscles and joints being used to scratch are not receiving signals directly from the itching skin. With cough, smooth muscle movement is incorporated into the actual cough reflex in order to move the irritant or blockage up the airway and out. It is this additional motility aspect that could result in more specialized involvement of myelinated fibers in cough, a specialization not needed in itch. Chronic cough is one of the most common reasons to visit the doctor and like chronic itch, can quickly become detrimental to quality of life [162]. Realizing the similarities between itch and cough can lead to new ideas and even perhaps, new ways to apply existing medications to new conditions.

Clinically, anti-histamines are often prescribed and have been shown to help with itch and cough. However, by no means do anti-histamines help with all conditions. This indicates a real need to discover the histamine-independent pathways involved. Progress has been made recently in histamine-independent itch research with the discovery of the family of Mrgprs.

Mrgpr genes encode a family of orphan G protein-coupled receptors (GPCRs) consisting of more than 50 members in the mouse genome [2,163,164]. The expression of many *Mrgpr*s, including *MrgprA3* and *MrgprC11*, is found in subsets of small-diameter sensory neurons in DRG and trigeminal ganglia [2,163,164]. The human *MrgprX*s is also selectively expressed in DRG neurons [165]. The specific expression pattern of Mrgprs in primary sensory neurons indicates that these receptors play essential roles in sensation such as pain and itch. Mrgprs should be examined for contributions to cough. Besides the DRG, MrgprA3 and MrgprC11 are also expressed in the mouse nodose/vagus ganglion, which innervates the airway. With the discovery of specific ligands for MrgprX1 and MrgprC11, such as BAM8-22, the tussive role of Mrgprs can be quickly explored. For example, β-alanine activates MrgprD^+^ neurons which are histamine insensitive [166]. It would be interesting to know whether application of Mrgpr agonists such as β-alanine can cause cough. If so, are Mrgpr expression levels increased under chronic cough conditions?

Realizing the similarities between itch and cough might result in the expansion of the repertoire of tussive agents. Or working in the other direction, increase the number of pruritogens, which is very useful for chemical probing. Expanding the knowledge of cough by realizing the parallels and similarities to itch can lead to new therapies and treatments for both. This increase of knowledge and theory could eventually lead to enhancements in treatments of chronic itch and cough that could help the millions suffering every day.

## Ethical approval

There are no unpublished experiments and data presented in this review article.

## Abbreviations

DRG: Dorsal root ganglia; RARs: Rapidly adapting receptors; SARs: Slowly adapting receptors; CMH: C fibers classified as mechanical and heat responsive; CMiHis+: Mechanically-insensitive C fibers that respond to histamine; PGE2: Prostaglandin E(2); CGRP: Calcitonin gene related peptide; TRPV1: Transient receptor potential vanilloid 1; GPCRs: G protein coupled receptors; PLC: Phospholipase C; DAG: Diacylglycerol; AD: Atopic dermatitis; SP: Substance P; NK1: Neurokinin 1; NK: Neurokinin; CFA: Complete freunds adjuvant; ACE: Angiotensin converting enzyme; CNPC: Chronic nonproductive cough; BAL: Bronchoalveolar lavage; UNDW: Ultrasonically nebulised distilled water; 5-HT: Serotonin; PAR: Protease activated receptor; GRPR: Gastrin-releasing peptide receptor; GRP: Gastrin-releasing peptide; HBE: Human bronchial epithelial.

## Competing interests

The authors declare that they have no competing interests.

## Authors’ contributions

PCL and XD wrote the manuscript. Both authors reviewed and approved the final manuscript.
